# Biological characterization, sequence type distribution and drug resistance profiling of *Mycoplasma hyorhinis* field isolates from pigs in Chongqing, China

**DOI:** 10.3389/fvets.2026.1732762

**Published:** 2026-01-30

**Authors:** Huiying Li, Xiuwu Lian, Yilin Li, Ruiyan Jin, Yunchong Ma, Ming Zhao, Yue Wu, Dongsheng Yi, Haixia Hu, Yujiao Yang, Honglei Ding

**Affiliations:** 1 Laboratory of Veterinary Mycoplasmology, College of Veterinary Medicine, Southwest University, Chongqing, China; 2 Chongqing Jiangbei Animal Disease Control Center, Chongqing, China; 3 Livestock and Aquaculture Technology Promotion Center of Beibei District, Chongqing, China

**Keywords:** 23S rRNA, growth curve, MLST, mlst, *Mycoplasma hyorhinis*

## Abstract

*Mycoplasma hyorhinis* is a ubiquitous pathogen of swine that causes polyserositis and polyarthritis and is also associated with conjunctivitis, meningitis, pneumonia, and abortions. This microorganism is a high prevalence pathogen in Chinese swine herds. However, few studies on *M. hyorhinis* have been reported in Chongqing, China. The overuse of antimicrobials has led to an increased risk of antimicrobial resistance, but a series of Chinese herbal monomers exhibited antibacterial activity to drug-resistant bacteria, including mycoplasmas. The aim of the study was to determine the prevalence, sequence types, growth kinetics, susceptibility to antimicrobials and Chinese herbal monomers, and relationships between the phenotypes and genotypes in terms of the resistance of *M. hyorhinis* to fluoroquinolones, macrolides and lincomycin. A total of 28 *M. hyorhinis* strains were recovered from the lungs of 404 slaughtered pigs. The isolates belonging to 11 novel STs, ST226, ST227, ST228, ST229, ST230, ST260, ST261, ST262, ST263, ST264 and ST265, were clustered separately from other reference isolates in the database. The growth kinetic of each isolate was generated, and the maximum color changing unit (CCU) values of isolates varied from 10^12^ to 10^20^ CCU/ml. *In vitro* susceptibility testing showed that the isolates were inhibited by low concentrations of tiamulin (MIC: ≤ 0.25 μg/ml), doxycycline (MIC: ≤ 0.25–1 μg/ml), ciprofloxacin (MIC: ≤ 0.25–2 μg/ml), florfenicol (MIC: 0.5–2 μg/ml), kanamycin (MIC: ≤ 0.25–4 μg/ml), enrofloxacin (MIC: 0.5–4 μg/ml) and berberine hydrochloride (MIC range: 1–8 μg/ml). However, the MICs of erythromycin were high (MIC: 32–≥128 μg/ml) for all isolates. The MICs of tylosin, tilmicosin and lincomycin for 50%, 60.7% and 46.4% of isolates were equal to or >16, 32 and 8 μg/ml, respectively. No correlation was detected between resistance to fluoroquinolones and QRDRs. However, the high MICs of tylosin, tilmicosin and lincomycin were most likely attributed to an A1553G mutation in domain V of the *23S rRNA*. Our findings demonstrated the diversity of STs among *M. hyorhinis* isolates in Chongqing. The high MICs of *M. hyorhinis* isolates to macrolides and lincomycin suggested that the use of lincomycin for the treatment of *M. hyorhinis* infections should be carefully evaluated.

## 1 Introduction

*Mycoplasma hyorhinis*, a species belonging to the Mollicutes class, is a small, fastidious, and cell wall-free bacterium that was first isolated in 1953 (1). For decades, it was widely regarded as a commensal bacterium that inhabits the ciliated upper respiratory tract of swine, with rare manifestation of overt clinical signs (2). However, under certain conditions, polyserositis and polyarthritis, are most frequently observed in infected pigs (3–5). The pathogen has also been implicated in conjunctivitis, otitis, meningitis, pneumonia and abortions (6, 7). Notably, *M. hyorhinis* has further been linked to multiple human malignancies, such as gastric, esophageal, lung, breast, glioma and colon cancers (8). In contrast to well-established research on other porcine *Mycoplasma* species such as *M. hyopneumoniae*, few studies of have employed molecular typing to characterize isolate heterogeneity, trace infection sources, or resolve phylogenetic relationships among *M. hyorhinis* strains.

A commercial vaccine against *M. hyorhinis* infection (Ingelvac MycoMAX™, Boehringer Ingelheim Animal Health, USA, Inc., Duluth, USA) is available. However, to date, its use is only authorized in the United States. Thus, beyond optimizing husbandry conditions for prevention, antimicrobial therapy remains the sole strategy for controlling active infections in affected animals. Due to their lack of a cell wall, *Mycoplasma* species are inherently resistant to antibiotics targeting cell wall biosynthesis, such as β-lactams, glycopeptides, and fosfomycin (9, 10). Additionally, the absence of key enzymes in the folate metabolic pathway renders sulfonamides and trimethoprim—agents that target this pathway—ineffective (11, 12). Despite these limitations, several antimicrobial classes are clinically useful for treating *M. hyorhinis* infections, though field isolates exhibit variable susceptibility profiles across studies (13–15).

Macrolides (e.g., tylosin, tilmicosin, tylvalosin) and fluoroquinolones (e.g., enrofloxacin, difloxacin) are the most widely prescribed antimicrobial families for metaphylaxis and treatment of *M. hyorhinis* infections globally, including in China (16). However, previous studies have documented a marked increase in minimal inhibitory concentrations (MICs) of field isolates to these agents (13–15, 17). Acquired resistance to macrolides and lincosamides arises via multiple mechanisms, including enzymatic inactivation of macrolides, enhanced efflux pump activity, and methylation or structural alteration of macrolide-binding sites on the ribosome (18).

Point mutations in domain V of the *23S rRNA* gene—a critical macrolide/lincosamide-binding region—have been well-documented in resistant *M. hyorhinis* strains (19–22). Furthermore, mutations in domain II of the *23S rRNA* gene, as well as in *rplD* and *rplV* (which encode ribosomal proteins L4 and L22, respectively), represent major mechanisms of macrolide resistance across *Mycoplasma* species (19–22). Molecular characterization of the quinolone resistance-determining regions (QRDRs) of DNA gyrase and topoisomerase IV in *M. hyorhinis* isolates with different levels of susceptibility to fluoroquinolones revealed that enrofloxacin-resistant isolates harbored amino acid substitutions in GyrA, ParC and ParE of the QRDRs (15). Importantly, mutation hotspots in these resistance-associated genes vary across drug-resistant strains, and numerous uncharacterized mutations may also contribute to antimicrobial resistance (19–22).

To date, few report of *M. hyorhinis* have been published in Chongqing Municipality, China. To address this knowledge gap, we isolated *M. hyorhinis* from swine lung tissues collected at local slaughterhouses and characterized the biological properties of these isolates. Specifically, we analyzed sequence type (ST) distribution, generated growth curves, and evaluated susceptibility to antimicrobials and Chinese herbal monomers. Additionally, we established correlations between phenotypic resistance profiles and underlying genotypic alterations for macrolides and fluoroquinolones.

## 2 Materials and methods

### 2.1 Sample collection and *M. hyorhinis* isolation

From March 30th to May 23rd, 2024, 404 lung specimens with confirmed pathological lesions were collected from slaughtered pigs at a slaughterhouse in Chongqing. These pigs were sourced from multiple distinct pig farms (Table 1). Prior to sample processing, the lung surface was disinfected with 3% hydrogen peroxide, after which a rice-grain-sized internal tissue fragment was homogenized in 1 ml of complete KM2 medium (KM2 broth medium containing 20% porcine serum and 0.01% NAD, pH 7.5) (23) supplemented with 100 μg/ml ampicillin. Homogenates were incubated at 37 °C for 7–14 days, with a red-to-yellow color change indicating medium acidification. After three passages, cultures were plated on KM2 agar and incubated at 37 °C for 5–10 days. Fried egg-like colonies were selected, expanded in complete KM2 broth, and re-plated on KM2 agar; this purification was repeated a minimum of three times to ensure monoclonality.

**Table 1:** Samples and isolates data.

**Sampling time**	**Pig farm**	**No. of samples**	**Isolates**	**Isolation rate**
March 30th, 2024	Pig farm 1	10	CQ001	10.0%
April 19th, 2024	Pig farm 2	43	CQ002, CQ003, CQ004	7.0%
April 23rd, 2024	Pig farm 3	22	CQ005	4.5%
	Pig farm 4	32	CQ024	3.1%
May 7th, 2024	Pig farm 5	43	CQ006, CQ007, CQ008, CQ009, CQ010, CQ011	14.0%
	Pig farm 6	18	CQ025, CQ026	11.1%
May 11th, 2024	Pig farm 7	27	CQ012	3.7%
May 16th, 2024	Pig farm 8	32	CQ013	3.1%
May 21st, 2022	Pig farm 9	45	CQ014, CQ015, CQ016, CQ017	8.9%
	Pig farm 10	26	CQ018, CQ019	7.7%
	Pig farm 11	34	CQ020, CQ021, CQ022	8.8%
	Pig farm 12	42	CQ027, CQ028	4.8%
May 23rd, 2024	Pig farm 13	30	CQ023	3.3%
Total		404		6.9%

DNA was extracted from the purified final culture using rapid boiling DNA extraction. Each broth sample was centrifuged at 13,500 × g for 10 min and discarded the supernatant. The bacterial pellet was resuspended in 50 μl of sterile double-distilled water. The suspended cells were treated with dry heat block at 105 °C for 5 min, placed on ice for 10 min, and centrifuged at 13,500 × g for 5 min. The supernatant containing the DNA template was collected. A real-time TaqMan PCR assay was conducted by amplification of *p37* gene of *M. hyorhinis* (24) using *ApexHF* HS DNA Polymerase FS Master Mix (Accurate Biology, Changsha, Hunan, China). Moreover, a partial sequence of the *16S rRNA* gene of purified final culture was amplified by PCR using a primer pair (forward:5′-ACCCGAGAACGTATTCAC3′; reverse:5′-GCAAGCGTTATCCGGA3′). The PCR was performed in a 25 μl reaction volume consisting of 0.5 μl of DNA template, 0.5 μl of each primer (10 μm), 12.5 μl of *ApexHF* HS DNA Polymerase FS Master Mix, and 11 μl of ddH_2_O. The amplification protocol consisted of an initial denaturation step at 94 °C for 5 min, followed by 30 cycles of amplification at 98 °C for 10 s, annealing at 55 °C for 10 s, and extension at 72 °C for 45 s, with a final extension at 72 °C for 5 min. The PCR products were subsequently sequenced in both directions by Sangon Biotech Co., Ltd.

### 2.2 Multilocus sequence typing (MLST) assay

MLST primers for six housekeeping genes were synthesized according to published methods (Supplementary Table S1) (25). PCR was performed in 25 μl reactions with *ApexHF* HS DNA Polymerase FS Master Mix, following the method of Trüeb et al. (25), and products were sequenced by Sangon Biotech (Shanghai, China). The DNA sequences of the six housekeeping genes were submitted to the PubMLST database (https://pubmlst.org/organisms/mycoplasma-hyorhinis) for allele IDs assignment, and an allelic profile (six loci) was generated for each strain to determine STs. STs were compared with 176 reference strains (from 9 countries across Asia, Europe, North America) in the database. BURST analysis (26), in which allelic profiles matched at 4 loci, was applied to divide the strains into various lineage groups. A phylogenetic tree was constructed by MEGA 12.0 and visualized and annotated using iTOL v7.2 (https://itol.embl.de/).

### 2.3 Growth kinetics of *M. hyorhinis* isolates

*M. hyorhinis* strains were inoculated into complete KM2 medium at a ratio of 1:9, followed by incubation at 37 °C. When the medium turned yellow, the culture was inoculated into two portions of fresh complete KM2 medium portion (1:9 ratio) and incubated at 37 °C. At 0 h, 12 h, 24 h, 36 h, 48 h, 60 h, 72 h, 84 h, and 96 h, 200 μl and 100 μl aliquots were collected from each culture. The optical density at 600 nm (OD_600_) of each 200 μl sample was measured using an ELISA plate reader (Thermo Fisher Scientific, Ratastie 2, FI-01620 Vantaa, Finland). Serial tenfold dilutions were performed on 100 μl samples: 100 μl sample was added to 900 μl complete KM2 medium, then 100 μl of this suspension was transferred to a second tube with 900 μl medium. Dilutions were continued from the 2nd to the 22nd tube, with the 23rd tube as a negative control. Tubes were incubated at 37 °C for 14 days; no visible growth (no red to yellow color change) was confirmed. The color changing unit (CCU) the bacterial fluid at each time point was the average of three repeats. Three growth-related curves were plotted for each isolate: (1) h-OD_600_ curve (*X*-axis: time points; *Y*-axis: average OD_600_ values), (2) h-lgCCU curve (*X*-axis: time points; *Y*-axis: lgCCU values), and (3) growth curve (*X*-axis: average OD_600_ values; *Y*-axis: lgCCU values).

### 2.4 *In vitro* susceptibility testing

The minimum inhibitory concentration (MIC) of *M. hyorhinis* isolates for 15 antimicrobials and 12 Chinese herbal monomers (Supplementary Table S2) was determined via broth microdilution (27). Kanamycin, gentamicin, amikacin, apramycin, chlortetracycline, doxycycline, tylosin, tylvalosin, lincomycin, tiamulin, enrofloxacin and ciprofloxacin were diluted with ddH_2_O at room temperature. Berberine hydrochloride was diluted with ddH_2_O at 40 °C. Florfenicol, erythromycin, tilmicosin, allicin, gingerenone A, baicalin, trans-cinnamic acid, cinnamaldehyde, gallic acid, glycyrrhizic acid, quercetin, curcumin, sodium houttuyfonate and ferulic acid were diluted with DMSO. The broth microdilution test was performed in 96-well microliter plates (31121, Labselect, Beijing, China) with two-fold serial dilutions (antimicrobials: 0.25–128 μg/ml; herbal monomers: 0.25–256 μg/ml) in 100 μl complete KM2 medium with sterility, pH and growth control. *M. hyorhinis* cultures (105 CCU/ml, 100 μl) were inoculated into wells and incubated at 35 ± 2 °C; MICs were read after 1 week (no further growth). All tests were run in triplicate. Notably, official breakpoint criteria for *in vitro* susceptibility testing of animal mycoplasmas are currently lacking. The *M. hyorhinis* reference strain BTS7 (ATCC 17981) was used as a quality control for MIC determination. MIC50 and MIC90 values were defined as the lowest concentrations inhibiting the growth of 50% and 90% of the tested strains, respectively (27).

### 2.5 PCR and nucleotide sequence analysis of QRDRs and domains II and V of the *23S rRNA* genes

PCR was used to detect the mutations within four QRDRs of *gyrA, gyrB, parC* and *parE*, and domains II and V of the *23S rRNA* genes to analyze the resistance mechanisms. The primer sequences and corresponding PCR amplification conditions are presented in Supplementary Table S1, as described in previous studies (15, 22). The PCR products were subjected to electrophoresis on a 1.5% agarose gel, and positive amplicons were bidirectionally sequenced. The sequencing results were aligned and analyzed using BLAST (http://www.ncbi.nlm.nih.gov/BLAST/). The resulting DNA sequences and the corresponding amino acid sequences of all the PCR products were compared with the *gyrA, parC* and *parE* sequences (accession number: NZ_KB911491) and the *23S rRNA* sequence (accession number: AB182581) of the *M. hyorhinis* BTS7 (ATCC 17981) strain and the *gyrB* sequence of the *M. hyorhinis* HUB-1 strain (accession number: CP002170).

## 3 Results

### 3.1 Isolation frequency of *M. hyorhinis* and MLST analysis

A total of 28 *M. hyorhinis* isolates were obtained from 404 lung samples (6.9%). *M. hyorhinis* isolates were confirmed by single colony selection, amplification of the *M. hyorhinis*-specific *p37* gene, and sequence alignment between the *16S rRNA* of the isolates and that of *M. hyorhinis* strains. After the nucleotide sequences were submitted to the PubMLST database for homology alignment, which revealed that none of the STs of the 28 isolates matched any entries in the database. Nucleotide sequences of the isolates were submitted to the PubMLST database for homology alignment, which revealed that none of the STs of the 28 isolates matched any entries in the database. These results indicated that the isolates identified in this study represent novel STs that have not been previously recorded in the database. Following reannotation by the database curator, the 28 isolates were classified into 11 distinct ST patterns (Tables 2, 3). Among these, ST226 and ST229 were the most prevalent, each accounting for 25.0% (7/28) of the total isolates. Three isolates (10.7%, 3/28) were assigned to ST227, while ST230, ST260, and ST262 each comprised two strains (7.1%, 2/28). Additionally, five isolates were distributed across five unique STs, namely ST228, ST261, ST263, ST264, and ST265.

**Table 2:** Sequence types of 28 *M. hyorhinis* isolates in this study.

**Sequence type**	**No. of *M. hyorhinis* isolates**
226	7 (25.0%)
227	3 (10.7%)
228	1 (3.6%)
229	7 (25.0%)
230	2 (7.1%)
260	2 (7.1%)
261	1 (3.6%)
262	2 (7.1%)
263	1 (3.6%)
264	1 (3.6%)
265	1 (3.6%)
Total	28

**Table 3:** Sequence types of *M. hyorhinis* isolates.

**Isolate**	***dnaA***	***rpoB***	***gyrB***	***gltX***	***adk***	***gmk***	**ST**
CQ001	26	1	3	4	1	5	228
CQ002	38	31	4	4	2	5	260
CQ003	10	3	3	4	2	3	229
CQ004	38	31	1	4	2	3	261
CQ005	38	31	4	4	2	5	260
CQ006	38	1	4	4	2	5	262
CQ007	1	1	1	4	2	1	226
CQ008	1	1	1	4	2	1	226
CQ009	1	1	1	4	2	1	226
CQ010	1	1	1	4	2	1	226
CQ011	1	1	1	4	2	1	226
CQ012	38	1	4	4	2	5	262
CQ013	2	3	18	4	15	5	263
CQ014	10	3	3	4	2	3	229
CQ015	10	3	3	4	2	3	229
CQ016	10	3	3	4	2	3	229
CQ017	10	1	4	4	2	5	230
CQ018	10	3	3	4	2	3	229
CQ019	10	1	4	4	2	5	230
CQ020	10	3	3	4	2	3	229
CQ021	1	3	11	4	8	3	227
CQ022	1	3	11	4	8	3	227
CQ023	1	1	11	4	8	3	265
CQ024	10	31	4	4	2	5	264
CQ025	1	1	1	4	2	1	226
CQ026	1	1	1	4	2	1	226
CQ027	10	3	3	4	2	3	229
CQ028	1	3	11	4	8	3	227

### 3.2 Alleles for housekeeping genes

Three novel alleles were identified among the isolates, designated as *dnaA-38* (5), *rpoB-31* (4), and *gyrB-18* (*n*). Specifically, three strains carried two novel gene sequences, whereas four strains harbored a single novel allelic variant. As summarized in Table 4, the *dnaA* gene exhibited five allelic variants: *dnaA-1* was the dominant allele, detected in 39.3% (11/28) of isolates, followed by *dnaA-10* (35.7%, 10/28), *dnaA-38* (17.9%, 5/28), *dnaA-2* (3.6%, 1/28), and *dnaA-26* (3.6%, 1/28). The *rpoB* gene was represented by three alleles: *rpoB-1* (46.4%, 13/28), *rpoB-3* (39.3%, 11/28), and *rpoB-31* (14.3%, 4/28). For the *gyrB* gene, five alleles were identified across the 28 strains: *gyrB-1* (28.6%, 8/28), *gyrB-3* (28.6%, 8/28), *gyrB-4* (25.0%, 7/28), *gyrB-11* (14.3%, 4/28), and *gyrB-18* (3.6%, 1/28). Notably, *gltX-4* was the sole allelic variant of the *gltX* gene detected in all isolates. Regarding the *adk* gene, *adk-2* was the most abundant allele (78.6%, 22/28), followed by *adk-8* (14.3%, 4/28), *adk-1* (3.6%, 1/28), and *adk-15* (3.6%, 1/28). For the *gmk* gene, *gmk-3* was the predominant allele (42.9%, 12/28), with *gmk-1* (25.0%, 7/28) and *gmk-5* (32.1%, 9/28) as the other two variants.

**Table 4:** Alleles of the 6 loci and their distribution.

**Gene**	**Allele**	**Frequency (%)**	**Number of alleles**	**Number of new alleles**	**New allelles**
*dnaA*	1	11 (39.3)	5	1	38
	2	1 (3.6)			
	10	10 (35.7)			
	26	1 (3.6)			
	38	5 (17.9)			
*rpoB*	1	13 (46.4)	3	1	31
	3	11 (39.3)			
	31	4 (14.3)			
*gyrB*	1	8 (28.6)	5	1	18
	3	8 (28.6)			
	4	7 (25.0)			
	11	4 (14.3)			
	18	1 (3.6)			
*gltX*	4	28 (100.0)	1	0	–
*adk*	1	1 (3.6)	4	0	–
	2	22 (78.6)			
	8	4 (14.3)			
	15	1 (3.6)			
*gmk*	1	7 (25.0)	3	0	–
	3	12 (42.9)			
	5	9 (32.1)			

### 3.3 BURST analysis

**Figure 1:**
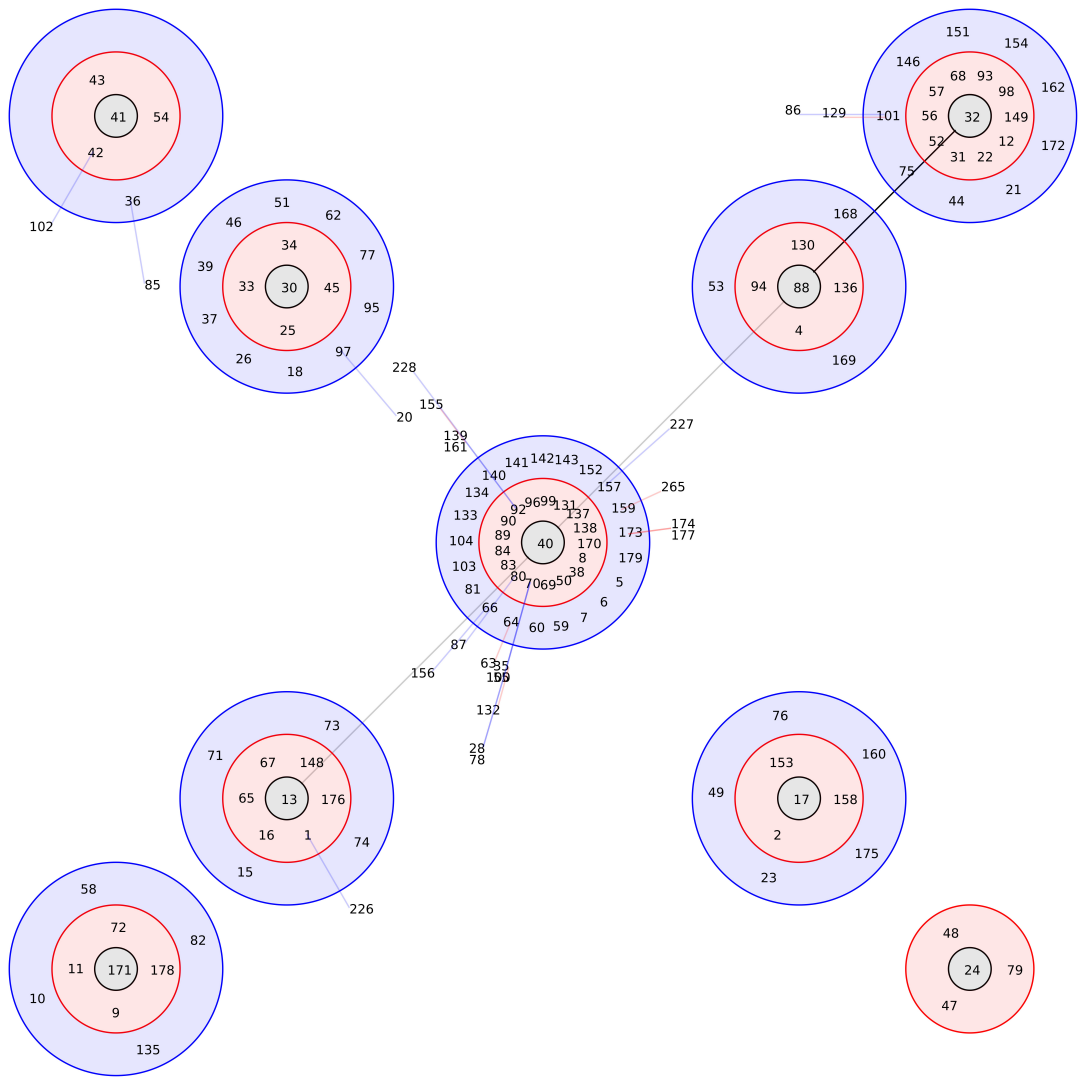
Graphic representations of the BURST analysis of group 1. The center genotype is located in the center circle. The single-locus variants (SLVs) are indicated by a red circle surrounding the central profile. The double-locus variants (DLVs) are shown as blue circles. The most distant profiles (triple-locus variants) are linked with a line.

To investigate the genetic clustering of the isolates, BURST analysis was performed with a threshold of 4 shared allelic loci among group members. A total of 204 STs were included in the analysis, consisting of the 28 STs identified in this study and 176 reference STs from domestic and international sources. Notably, all STs were clustered into one group (data not shown). ST40 was identified as the central genotype, with ST13, ST17, ST24, ST30, ST32, ST41, ST88, and ST171 forming eight distinct subgroups (Figure 1). Sequence divergence analysis showed that ST40 differed from ST265 at 3 loci and from ST227 and ST228 at 4 loci. ST13 differed from ST226 at 3 loci. Seven additional STs were not visualized in Figure 1, as these did not cluster within any of the eight established subgroups. The inability of these isolates to form independent subgroups separate from other isolates is likely due to the insufficient data present in the current database.

**Figure 2:**
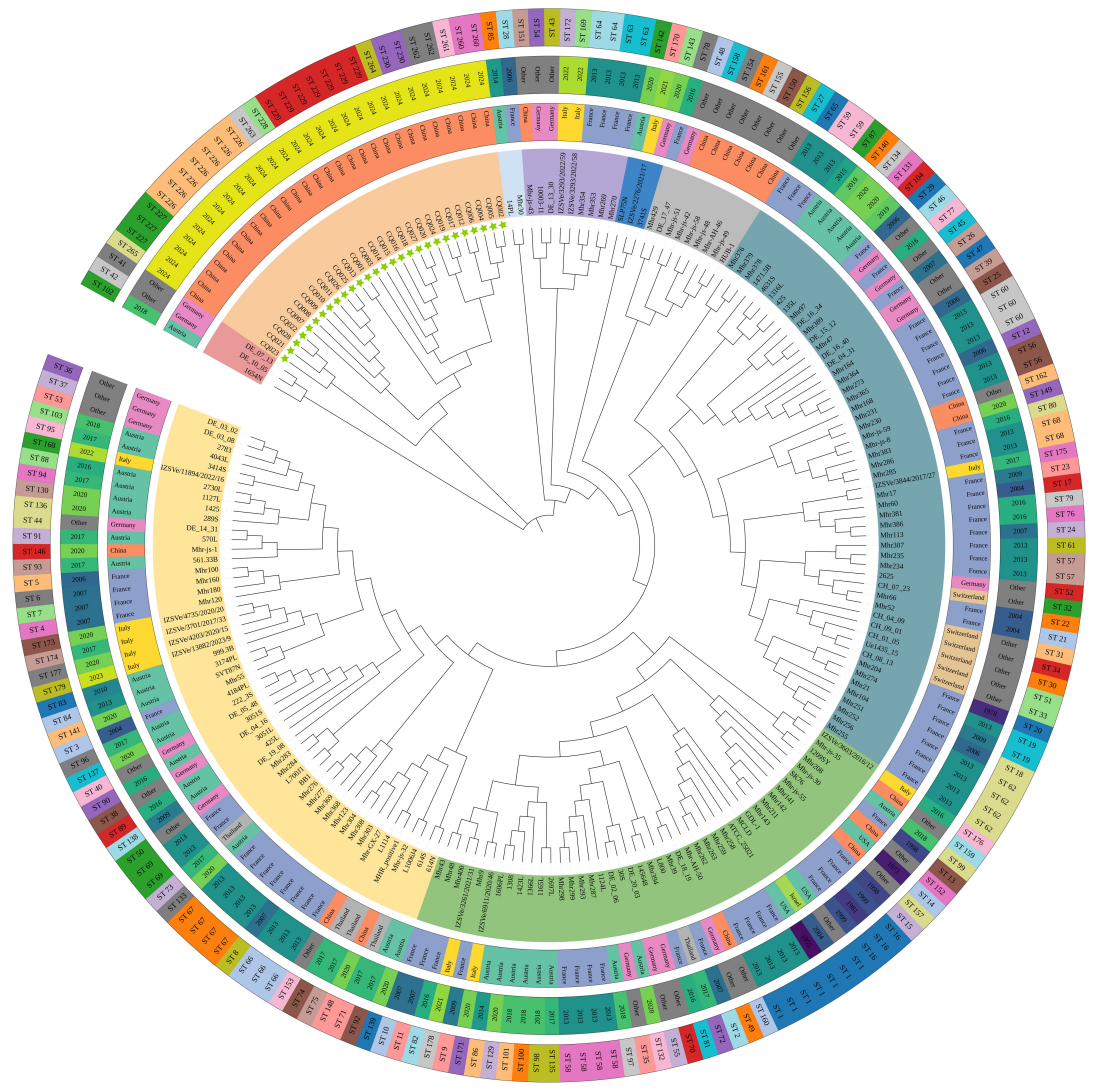
MLST neighbor-joining tree of 204 *M. hyorhinis* isolates belonging to 158 STs. The phylogenetic tree was developed with the MEGA 12.0 program and visualized via the iTOL v7.2 program (https://itol.embl.de/). The rings (starting from the innermost) contain information on isolate identifiers; the green pentagrams indicate the strains isolated in this study, and the different background colors of the isolates represent different groups. The three outer rings from inside to outside are country, year and ST type.

Furthermore, a phylogenetic tree was constructed based on the concatenated nucleotide sequences of six housekeeping genes using the neighbor-joining method (Figure 2). The 28 isolates from this study, marked with green pentagrams, formed a distinct clade that was separated from all reference isolates in the database.

### 3.4 Growth curves of the isolates

**Figure 3:**
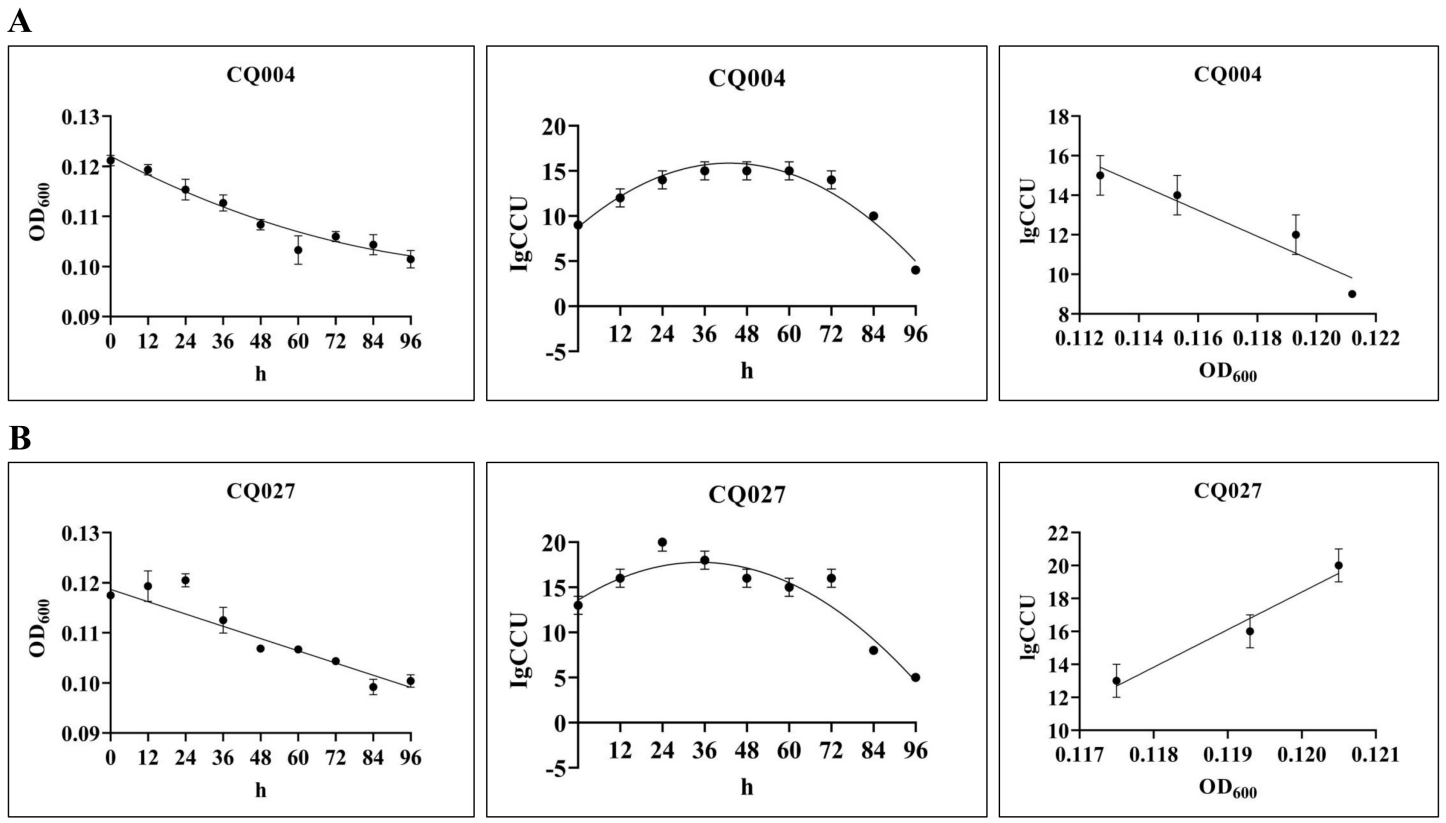
The h-OD_600_ curves, h-lgCCU curves and growth curves of *M. hyorhinis* isolates CQ004 and CQ027.

The h-OD_600_ curves, h-lgCCU curves, and growth curves of all isolates are presented in Table 5, Figure 3, Supplementary Figures S1, S2. Overall, the OD600 values of isolates CQ001–CQ024, CQ026, and BTS07 exhibited a gradual decline with prolonged cultivation time. In contrast, isolates CQ025, CQ027, and CQ028 showed an increase in OD600 values up to 24 h of cultivation, followed by a gradual decrease thereafter. With respect to lgCCU, all isolates displayed a phase of increase until a specific time point, after which lgCCU values declined. The peak lgCCU was observed at 24 h for isolates CQ001, CQ003, CQ010, CQ012, CQ016, CQ021, and CQ026–CQ028; at 36 h for isolates CQ004–CQ006, CQ008, CQ009, CQ011, CQ013–CQ015, CQ017, CQ020, CQ023–CQ025, and BTS07; and at 48 h for isolates CQ002, CQ007, CQ018, CQ019, and CQ022. Growth curves were plotted with the average OD600 values at 0 h, 12 h, 24 h, 36 h, and 48 h on the *X*-axis and the corresponding lgCCU values on the *Y*-axis. As shown in Figure 3, Supplementary Figures S1, S2, a negative correlation between OD600 and lgCCU values was observed for isolates CQ001–CQ024, CQ026, and BTS07. In contrast, isolates CQ025, CQ027, and CQ028 exhibited a positive correlation between these two parameters during the 0–24 h cultivation period. Significant inter-isolate variability was observed in the maximum CCU achieved in complete KM2 medium, with values ranging from 10^2^^2^ to 10^2^0 CCU/ml (Table 5).

**Table 5:** Growth curves of *M. hyorhinis* isolates.

**Isolate**	**Growth curve**	**Maximum value of CCU (CCU/ml)**
CQ001	*Y* = −1,561.6*X* + 198.76	10^16^
CQ002	*Y* = −411.71*X* + 57.261	10^12^
CQ003	*Y* = −949.83*X* + 124.96	10^16^
CQ004	*Y* = −656.53*X* + 89.396	10^15^
CQ005	*Y* = −471.7*X* + 69.409	10^15^
CQ006	*Y* = −285.51*X* + 48	10^16^
CQ007	*Y* = −479.8*X* + 70.335	10^14^
CQ008	*Y* = −135.55*X* + 26.648	10^12^
CQ009	*Y* = −1,233.3*X* + 157.83	10^15^
CQ010	*Y* = −212.5*X* + 34.427	10^12^
CQ011	*Y* = −403.25*X* + 61.646	10^16^
CQ012	*Y* = −5,303.9*X* + 649.78	10^14^
CQ013	*Y* = −441.05*X* + 66.147	10^16^
CQ014	*Y* = −380.73*X* + 58.819	10^16^
CQ015	*Y* = −520*X* + 74.18	10^16^
CQ016	*Y* = −614.73*X* + 85.65	10^16^
CQ017	*Y* = −640*X* + 88.393	10^16^
CQ018	*Y* = −728.74*X* + 96.784	10^16^
CQ019	*Y* = −744.98*X* + 96.302	10^13^
CQ020	*Y* = −314.63*X* + 46.592	10^13^
CQ021	*Y* = −1,231.4*X* + 158.68	10^16^
CQ022	*Y* = −787.23*X* + 105.16	10^16^
CQ023	*Y* = −339.08*X*+ 53.925	10^15^
CQ024	*Y* = −766.67*X* + 105.65	10^16^
CQ025	*Y* = 1,277.8*X* – 136.28	10^16^
CQ026	*Y* = −2,720.6*X* + 337.67	10^18^
CQ027	*Y* = 2,271.3*X* – 254	10^20^
CQ028	*Y* = 1,578.9*X* – 174.26	10^20^
BTS07	*Y* = −476.19*X* + 67.167	10^14^

### 3.5 Susceptibility profiles of *M. hyorhinis* isolates to antimicrobial agents and Chinese herbal monomers

The MIC values, as well as the MIC50 and MIC90 values, for all tested antimicrobials and Chinese herbal monomers are summarized in Table 6 and Supplementary Table S3. The isolates demonstrated high susceptibility to tiamulin (MIC ≤ 0.25 μg/ml), doxycycline (MIC ≤ 0.25–1 μg/ml), ciprofloxacin (MIC ≤ 0.25–2 μg/ml), florfenicol (MIC 0.5–2 μg/ml), kanamycin (MIC ≤ 0.25–4 μg/ml), and enrofloxacin (MIC 0.5–4 μg/ml). The MICs of gentamicin (96.4%, 27/28), chlortetracycline (92.9%, 26/28), tylvalosin (85.7%, 24/28) and apramycin (82.1%, 23/28) for most of the isolates were lower than 8 μg/ml. In contrast, all isolates exhibited high MIC values (32–≥128 μg/ml) to erythromycin. A bimodal distribution of MIC values was observed for tylosin and tilmicosin: 50.0% (14/28) of isolates had a tylosin MIC ≤ 1 μg/ml, and 39.3% (11/28) of isolates had a tilmicosin MIC ≤ 2 μg/ml.

**Table 6:** Summary of the MIC range and MIC_50_ and MIC_90_ values (μg/ml) of the *M. hyorhinis* isolates for various antimicrobial agents and Chinese herbal monomers.

**Antimicrobial class**	**Antimicrobial/Chinese herbal monomer**	**MIC**											**MIC_50_**	**MIC_90_**
		≤ **0.25**	**0.5**	**1**	**2**	**4**	**8**	**16**	**32**	**64**	**128**	≥**256**		
Aminoglycosides	Kanamycin	6	3	4	10	5							2	4
	Gentamicin	4	7	3	6	7	1						2	4
	Amikacin			1	5	3	1	10	7	1			16	32
	Apramycin		1	4	9	9	1	4					2	16
Tetracyclines	Chlortetracycline		5	5	6	10	2						2	4
	Doxycycline	16	11	1									≤ 0.25	0.5
Amphenicols	Florfenicol		20	5	3								0.5	2
Macrolides	Erythromycin								2	17	9		64	128
	Tylosin	13		1				1	5	6	2		1	64
	Tylvalosin	14	1	1	5	3	4						0.5	8
	Tilmicosin	2	7	1	1				4	11	2		32	64
Lincosamides	Lincomycin	5	3	5	2		1		1	5	6		2	128
Pleuromutilin	Tiamulin	28											≤ 0.25	≤ 0.25
Fluoroquinolones	Enrofloxacin		8	13	3	4							1	4
	Ciprofloxacin	18	5	4	1								≤ 0.25	1
	Berberine hydrochloride			4	6	14	4						4	8
	Allicin							10	11	7			32	64
	Gingerenone A							3	11	8	6		32	128
	Baicalin									4	24		128	128
	Trans-cinnamic acid								6	9	10	3	64	≥256
	Cinnamaldehyde									4	20	4	128	≥256
	Gallic acid									3	19	6	128	≥256
	Glycyrrhizic acid									3	23	2	128	128
	Quercetin									5	22	1	128	128
	Curcumin									6	12	10	128	≥256
	Sodium houttuyfonate									1	21	6	128	≥256
	Ferulic acid										15	13	128	≥256

Overall, compared with antimicrobials, most Chinese herbal monomers exhibited higher MIC values against *M. hyorhinis* isolates (Table 6, Supplementary Table S3). The MIC ranges of the tested herbal monomers were as follows: ferulic acid (128 to ≥256 μg/ml); cinnamaldehyde, gallic acid, glycyrrhizic acid, quercetin, curcumin, and sodium houttuyfonate (64 to ≥256 μg/ml); trans-cinnamic acid (32 to ≥256 μg/ml); baicalin (64–128 μg/ml); gingerenone A (16–128 μg/ml); and allicin (16– 64 μg/ml). Notably, the MICs of 85.7% (24/28) of the isolates for berberine hydrochloride were < 8 μg/ml.

### 3.6 Molecular characterization of the QRDRs of *M. hyorhinis* isolates

All isolates were phenotypically susceptible to the two tested fluoroquinolones, although the MIC values of enrofloxacin for four isolates were equal to 4 μg/ml. To explore the potential association between QRDR mutations and MIC values, we amplified four genes encoding QRDRs by using the primers designed by Li et al. (15). PCR amplification results showed that *gyrA* and *parE* were successfully amplified in all 28 isolates, whereas *gyrB* and *parC* were detected in only 8 and 23 isolates, respectively.

Nucleotide sequence alignment was performed using the *gyrA, parC*, and *parE* sequences of the BTS7 strain and the *gyrB* sequence of the HUB-1 strain as references. Several nonsynonymous mutations were identified in one or two QRDR genes across the isolates (Table 7, Supplementary Table S3). A conserved nucleotide substitution at position 185 (A185G) in the *gyrA* gene resulted in an I62S amino acid substitution in all the strains. A nucleotide mutation at position 274 (A274G) in the *parC* gene led to an I92V amino acid change in 5 strains. For the *parE* gene, a nonsynonymous mutation at position 1098 (C1098A) caused an E366D amino acid substitution in 6 strains; additionally, isolate CQ005 carried two mutations in *parE* (T873A and C1098A), which resulted in amino acid substitutions N291K and E366D, respectively. No mutations were detected in the amplified *gyrB* gene fragments.

**Table 7:** Prevalence of QRDRs in *M. hyorhinis* isolates.

**Combination of QRDRs**			**Total**
**GyrA**	**ParC**	**ParE**	
I62S			16
I62S	I92V		5
I62S		E366D	6
I62S		N291K, E366D	1

Notably, none of the mutations identified in this study matched those previously reported by Li et al. (15). To further explore genetic variations in *gyrB*, we aligned the *gyrB* sequences used for MLST analysis (25) against the HUB-1 reference sequence. A nonsynonymous mutation at position 1708 (G1708A) was detected in isolates CQ021, CQ022, and CQ023, leading to a V570I substitution. Collectively, these results indicate that no key QRDR mutations in *gyrA, gyrB, parC*, or *parE* were associated with elevated enrofloxacin or ciprofloxacin MIC values in the tested isolates.

### 3.7 Mutational analysis of domains II and V of the *23S rRNA* in *M. hyorhinis* isolates

Nucleotide sequence alignment of the *23S rRNA* gene against the BTS7 reference strain revealed no mutations in domain II among the 28 isolates. In contrast, two distinct mutations were identified in domain V: an A1553G substitution was detected in 15 isolates, and a C2014T substitution was uniquely present in isolate CQ013 (Supplementary Table S3). The A1553G mutation appeared to be strongly correlated with high MIC values for tylosin, tylvalosin, tilmicosin, and lincomycin, despite the fact that all isolates exhibited high MICs of erythromycin (MIC: 32–≥128 μg/ml). Specifically, all isolates carrying the A1553G mutation displayed elevated MIC values for tylosin (≥16 μg/ml), tylvalosin (≥2 μg/ml), tilmicosin (≥32 μg/ml), and lincomycin (≥32 μg/ml), with three exceptions: isolate CQ017 showed low tylosin susceptibility (MIC ≤ 0.25 μg/ml), while isolates CQ003 and CQ004 exhibited low tylvalosin MIC values (0.5 and 1 μg/ml, respectively).

The mutation at A1553G of the *23S rRNA* in *M. hyorhinis* was equivalent to the mutation at A2058G on the basis of *23S rRNA* of *E. coli* (22). The C2014T mutation in domain V of the *23S rRNA* in the CQ013 strain was related at least to the high MICs to tilmicosin, as the MIC for CQ013 (MIC: 32 μg/ml) was greater than that for strains without mutation in domain V of the *23S rRNA* (MIC: ≤ 0.25 μg/ml) to tilmicosin.

## 4 Discussion

*M. hyorhinis* is distributed worldwide, with a high estimated prevalence (28, 29). On one hand, the pathogen can induce serious systemic inflammation, resulting in substantial economic losses to the pig industry (30); on the other hand, infected pigs often lack obvious clinical manifestations, and subclinical infections can may lead to growth retardation (29). Additionally, *M. hyorhinis* frequently coinfects with other swine pathogens, which exacerbates disease progression, increases mortality rates, and ultimately causes greater economic losses (7, 31). Therefore, the economic impact of *M. hyorhinis* infection should not be underestimated. Despite its significance, research on *M. hyorhinis* remains limited compared to that on *M. hyopneumoniae*.

*M. hyorhinis* typically exists as a commensal bacterium in the respiratory tract of pigs. In this study, we aimed to isolate *M. hyorhinis* from healthy pigs awaiting slaughter, therefore selecting porcine lung tissues as the isolation source. A total of 28 *M. hyorhinis* strains were successfully isolated using KM2 medium, instead of Friis broth—a conventional culture medium for mycoplasmas (32). KM2 broth is composed of lactalbumin hydrolysate (5 g/L), fresh yeast extract (10 g/L), Eagle's medium (5.975 g/L), Dulbecco's phosphate-buffered saline (2.925 g/L), and phenol red (0.007 g/L), and is widely used for the cultivation of *M. hyopneumoniae* (23, 33). Previous studies have also confirmed its applicability for *M. hyorhinis* culture (15, 34). Similar to other mycoplasma culture media, phenol red serves as a pH indicator in KM2 medium (35). During *M. hyorhinis* cultivation, the medium color changes from red to yellow, reflecting a shift in pH from weakly alkaline to acidic due to bacterial metabolic activities.

To establish a quantitative correlation between culture chromaticity and bacterial cell density, we characterized the growth kinetics of all isolates. The obtained specific growth curves enable accurate inference of bacterial load in cultures from the OD values of bacterial suspensions, thereby facilitating various downstream experiments. For instance, *in vitro* susceptibility testing, predefined growth curves allowed efficient harvesting of bacteria at a standardized concentration. Notably, the OD600 values of isolates CQ025, CQ027 and CQ028 increased from 0 to 24 h of cultivation and then gradually decreased, whereas the OD600 values of other strains consistently declined with prolonged culture time. This decrease in OD600 values may be attributed to nutrient consumption and metabolic changes that affect medium turbidity. After 24–48 h of cultivation, the lgCCU values of all strains decreased, indicating depletion of essential nutrients in the medium. To avoid the impact of nutrient exhaustion on growth curve characterization, we constructed growth curves for different time windows (0–24 h, 0–36 h, or 0–48 h) based on the growth characteristics of individual isolates.

To date, the *M. hyorhinis* PubMLST database has accumulated over 200 isolates and more than 150 STs. Among these, 17 strains from Chinese mainland are assigned to ST27, ST146, and ST148–ST162. In the present study, the combination of 6 MLST housekeeping gene alleles from each isolate did not match any existing STs in the database. Eventually, 11 novel ST patterns identified from the 28 isolates were submitted to and validated by the PubMLST database curators. Furthermore, significant variations were observed in the allelic diversity of the target housekeeping genes: 5 distinct alleles were detected for both *dnaA* and *gyrB*, while only one allele (*gltX-4*) was identified for *gltX*. In contrast, the reference database currently documents 38, 18, and 26 established alleles for *dnaA, gyrB*, and *gltX*, respectively. Notably, among the 6 MLST housekeeping genes, *gyrB*—which has the lowest number of known alleles in the PubMLST database—exhibited a relatively high allelic diversity (13.1%) in our isolates. Conversely, *gltX*, which has the largest number of curated alleles in the database, showed remarkably low allelic coverage (3.8%) in the tested strains. These findings collectively indicate that *M. hyorhinis* strains isolated from different geographic regions exhibit substantial genetic heterogeneity.

Previous studies from European countries have reported that *M. hyorhinis* strains have developed resistance to several macrolides and lincosamides, such as tylosin, tilmicosin, and lincomycin (13, 14, 36). Additionally, strains isolated from Italy showed reduced susceptibility to tiamulin (36). A domestic study in China also reported high MIC values of tylosin, tilmicosin, and lincomycin for 25 field isolates of *M. hyorhinis* (15). In the present research, we determined the MIC values of 14 veterinary antimicrobials (approved for swine use) against the isolates to screen for potential effective agents. Our results showed that most isolates exhibited high MICs for tylosin and tilmicosin, which may be associated with the widespread use of these antimicrobials in the pig industry. However, all tested isolates were susceptible to low concentrations of tiamulin (MIC ≤ 0.25 μg/ml), which contradicts the findings of Rosales et al. (36). This discrepancy may be attributed to differences in geographic origin, isolation time, or local antimicrobial use practices among the tested strains.

Enrofloxacin, a commonly used fluoroquinolone in swine production, is widely employed for the prevention and treatment of bacterial diseases, including *Mycoplasma* infections. Fifteen *M. hyorhinis* isolates collected in the United States between 1995 and 1998 showed a low MIC for enrofloxacin (37). In contrast, a study conducted across five European countries reported that moderate concentrations of enrofloxacin inhibited the growth of most of the 76 *M. hyorhinis* isolates collected between 2019 and 2021, with only one Hungarian isolate showing reduced susceptibility (14), suggesting a potential trend of increasing enrofloxacin MICs in more recently isolated strains. In our study, the MICs of enrofloxacin for the strains were relatively lower compared to those of other tested antimicrobials. Nevertheless, we investigated whether the high MIC value (4 μg/ml) of enrofloxacin observed in four isolates was associated with mutations in QRDRs. Li et al. (15) reported that mutations in ParC (Ser80Phe, Ser80Tyr, Phe80Tyr, Glu84Gly, and Glu84Lys), GyrA (Ala83Val, Ser84Pro, Asp87Tyr, and Asp87Asn), and ParE (Glu470Lys) were strongly associated with increased fluoroquinolone MICs. However, in our study, four strains whose MICs were 4 μg/ml for enrofloxacin showed mutations only in GyrA (I62S), while no mutations were detected in the other three proteins (or one or two genes could not be amplified). Mutations in GyrA were detected in all the strains. Considering that the MICs of enrofloxacin were low for the isolates, the mutations in the four PMQR proteins are not associated with enrofloxacin resistance in the tested strains.

Antimicrobial susceptibility testing revealed that all isolates exhibited high erythromycin MICs (32–≥128 μg/ml). Additionally, 50% (14/28) and 60.7% (17/28) of the isolates had tylosin and tilmicosin MICs ≥16 μg/ml and ≥32 μg/ml, respectively. A previous study indicated that a completely tylosin-resistant BTS7 mutant strain presented one point mutation at position A2059G, whereas the BTS7 mutant strain, which developed lincomycin resistance, presented two point mutations at positions G2597U and C2611U in domain V and the addition of an adenine at the pentameric adenine loop in domain II (22). Our results demonstrated that 15 isolates with an A1553G mutation in domain V of the *23S rRNA* had high MICs for tylosin (MIC: ≥ 16 μg/ml), tylvalosin (MIC: ≥ 2 μg/ml), tilmicosin (MIC: ≥ 32 μg/ml) and lincomycin (MIC: ≥ 32 μg/ml), except for CQ017, whose MICs were low for tylosin ( ≤ 0.25 μg/ml), and CQ003 (0.5 μg/ml) and CQ004 (1 μg/ml) yielded low MICs for tylvalosin. Therefore, the A1553G mutation was closely related to high MICs to tylosin, tylvalosin, tilmicosin and lincomycin. These results broaden the understanding of the resistance mechanism of *M. hyorhinis* to macrolides and lincosamides.

With respect to aminoglycosides, 67.9% (19/28) and 50.0% (14/28) of the isolates had amikacin and apramycin MICs > 4 μg/ml and >2 μg/ml, respectively. However, the resistance mechanisms of mycoplasmas to aminoglycosides have not been reported in the literature, warranting further investigation to elucidate the underlying genetic and biochemical pathways.

The widespread use of antimicrobials in food-producing animals has become a global concern. In response, the Chinese government launched the “National Action Plan for the Reduction of Veterinary Antimicrobial Use (2021–2025)” in 2021, emphasizing the need to promote the safe, standardized, and scientific use of veterinary antimicrobials, curb bacterial resistance, and support the development of alternative products (Ministry of Agriculture and Rural Affairs of the People's Republic of China, 2021). Several studies have reported the antibacterial or bacteriostatic effects of Chinese herbal monomers (38–41). In this study, we evaluated the antibacterial activity of several Chinese herbal monomers against *M. hyorhinis* isolates. Our results showed that most Chinese herbal monomers exhibited high MICs against the isolates, making them unsuitable as direct substitutes for antimicrobials. However, berberine hydrochloride—an extensively validated antibacterial Chinese herbal monomer—exhibited relatively low MICs (1–8 μg/ml) compared to other tested herbal monomers. These findings suggest that berberine hydrochloride holds great promise as a potential alternative to antimicrobials for controlling *M. hyorhinis* infections, aligning with the national strategy of reducing veterinary antimicrobial use.

## 5 Conclusion

In summary, we isolated 28 *M. hyorhinis* strains from the lungs of slaughtered pigs. These isolates belonged to 11 new STs. Tiamulin, doxycycline, ciprofloxacin, florfenicol, kanamycin, enrofloxacin and berberine hydrochloride were the most effective agents against the *M. hyorhinis* isolates *in vitro*, and erythromycin displayed high MICs for all the isolates. Moreover, the MICs of tylosin, tilmicosin and lincomycin for 50%, 60.7% and 46.4% of the isolates were equal to or >16 μg/ml, 32 μg/ml and 8 μg/ml, respectively. The high MICs of tylosin, tilmicosin and lincomycin were most likely attributed to an A1553G mutation in domain V of the *23S rRNA*.

## Data Availability

The original contributions presented in the study are included in the article/Supplementary material, further inquiries can be directed to the corresponding authors.

## Supplementary material

Supplementary Figure S1 The h-OD_600_ curves, h-lgCCU curves and growth curves of 13 *M. hyorhinis* isolates CQ001–CQ003 and CQ005–CQ014.

Supplementary Figure S2 The h-OD_600_ curves, h-lgCCU curves and growth curves of 13 *M. hyorhinis* isolates CQ015–CQ026 and CQ028.

Supplementary Table S1 Primers and PCR amplification protocols used in this study.

Supplementary Table S2 Antimicrobials and Chinese herbal monomers used in this study.

Supplementary Table S3 *In vitro* susceptibility to antimicrobials and Chinese herbal monomers and the molecular characterization of QRDRs, domains II and V of the *23S rRNA*.
