# Supplementary figures and images for: Biological characterization, sequence type distribution and drug resistance profiling of Mycoplasma hyorhinis field isolates from pigs in Chongqing, China
Source: Front Vet Sci. 2026 Jan 30;13:1732762. doi: 10.3389/fvets.2026.1732762 (PMC12902949; doi:10.3389/fvets.2026.1732762)

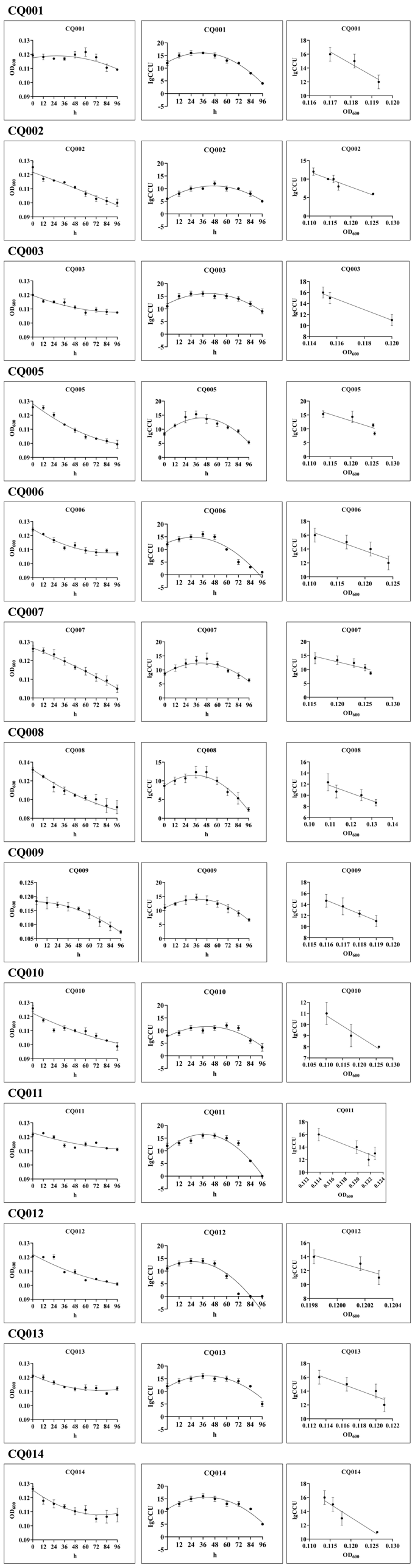

Supplement: Supplementary Figure S1 — The h-OD600 curves, h-lgCCU curves and growth curves of 13 M. hyorhinis isolates CQ001–CQ003 and CQ005–CQ014. [file Image_1.JPEG]

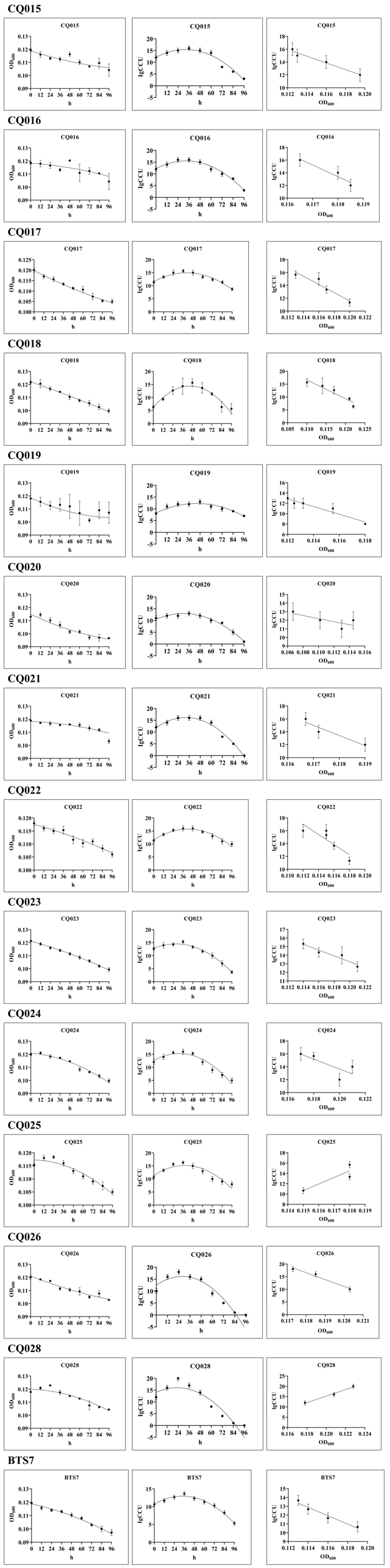

Supplement: Supplementary Figure S2 — The h-OD600 curves, h-lgCCU curves and growth curves of 13 M. hyorhinis isolates CQ015–CQ026 and CQ028. [file Image_2.JPEG]
