# Supplementary material for: Biological characterization, sequence type distribution and drug resistance profiling of Mycoplasma hyorhinis field isolates from pigs in Chongqing, China
Source: Front Vet Sci. 2026 Jan 30;13:1732762. doi: 10.3389/fvets.2026.1732762 (PMC12902949; doi:10.3389/fvets.2026.1732762)
Supplement: Supplementary Table S1 — Primers and PCR amplification protocols used in this study. [file Table_1.docx]

Supplementary Material

**TABLE** **S1.** Primers and PCR amplification protocols used in this study.

| Target gene | Primer name | Sequence (5'-3') | Product size | Annealing temperature | Reference |
| --- | --- | --- | --- | --- | --- |
| *dnaA* | dnaA-F | CAGAAGTCTTAGGTGGTTTTG | 459 bp | 55 ℃ | Trüeb et al., 2016 |
|  | DnaA-R | TGTGGAATGATCCTTGCCTC |  |  |  |
| *rpoB* | rpoB-F | TCAAGCTGTTCCATTAATTACTAC | 509 bp | 55 ℃ | Trüeb et al., 2016 |
|  | rpoB-R | GCACTAACTTCTGATCCAATAC |  |  |  |
| *gyrB* | gyrB-F | GATTCTGATGGTTCACATATTAG | 358 bp | 55 ℃ | Trüeb et al., 2016 |
|  | gyrB-R | GTCTAGGTTTTTTGCATATTTTGC |  |  |  |
| *gltX* | gltX-F | CTGAAAGACTCTCAAAATCACC | 448 bp | 55 ℃ | Trüeb et al., 2016 |
|  | gltX-R | TTACAAGCCTTTTTGAAATTAGTTC |  |  |  |
| *adk* | adk-F | CGATGGCATCTAATTCTTTTAAAG | 437 bp | 55 ℃ | Trüeb et al., 2016 |
|  | adk-R | TACTCAGGCAAAGTTTTTAGAAC |  |  |  |
| *gmk* | gmk-F | GCGCCTGTTTCTGTTAATATTG | 452 bp | 55 ℃ | Trüeb et al., 2016 |
|  | gmk-R | AAGAGACAAAAGACCTAATGAAG |  |  |  |
| *gyrA* | gyrA-F | ACTTCTTTTAAATTATGAGGG | 621 bp | 56 ℃ | Li et al., 2016 |
|  | gyrA-R | TGAAGCAGAACTAGAACAA |  |  |  |
| *gyrB* | gyrB-F | AATTAAACATTCAAATCCAATT | 670 bp | 56 ℃ | Li et al., 2016 |
|  | gyrB-R | TGAATATGCATAAACAACTT |  |  |  |
| *parC* | parC-F | ATGAAGAAACTAGATAATAATATG | 609 bp | 56 ℃ | Li et al., 2016 |
|  | parC-R | TTCTATACAAGCATCAATTA |  |  |  |
| *parE* | ParE-F | CACAGATAGTTATTCTGATTC | 831 bp | 56 ℃ | Li et al., 2016 |
|  | parE-R | GGTTGAGCTATGTAAACAT |  |  |  |
| domains II of *23S rRNA* | mhr-D2F | ATCCATGAGCAGGTTGAAGC | 193 bp | 60 ℃ | Kobayashi et al., 2005 |
|  | mhr-D2R | CCATTCCACATTCAGTGCTC |  |  |  |
| domains V of *23S rRNA* | mhr-D5-1F | CACGAAAGGCGCAATGATCTC | 192 bp | 60 ℃ | Kobayashi et al., 2005 |
|  | mhr-D5-1R | CACTAGAACTAGCGTCCCAGC |  |  |  |
|  | mhr-D5-2F | CTCATCGCATCCTGGAGCTG | 253 bp | 60 ℃ | Kobayashi et al., 2005 |
|  | mhr-D5-2R | CCGCTTAGATGCTTTCAGCG |  |  |  |

**TABLE** **S2.** Antimicrobials and Chinese herbal monomers used in this study.

| Antimicrobial or Chinese herbal monomer | Source | Identifier |
| --- | --- | --- |
| Kanamycin | Sangon Biotech, Shanghai, China | Cat # A600286 |
| Doxycycline | Macklin, Shanghai, China | Cat # D832390 |
| Amikacin | Macklin, Shanghai, China | Cat # A837351 |
| Apramycin | Macklin, Shanghai, China | Cat # A800502 |
| Lincomycin | Macklin, Shanghai, China | Cat # L107359 |
| Chlortetracycline | OriLeaf, Shanghai, China | Cat # S17011 |
| Tylvalosin | OriLeaf, Shanghai, China | Cat # S27405 |
| Tilmicosin | OriLeaf, Shanghai, China | Cat # S80304 |
| Tylosin | OriLeaf, Shanghai, China | Cat # S61369 |
| Erythromycin | OriLeaf, Shanghai, China | Cat # S17002 |
| Tiamulin | OriLeaf, Shanghai, China | Cat # S24754 |
| Enrofloxacin | OriLeaf, Shanghai, China | Cat # S26099 |
| Gentamicin | OriLeaf, Shanghai, China | Cat # S17024 |
| Florfenicol | OriLeaf, Shanghai, China | Cat # S17082 |
| Ciprofloxacin | OriLeaf, Shanghai, China | Cat # B24370 |
| Baicalin | Macklin, Shanghai, China | Cat # B802695 |
| Berberine hydrochloride | Macklin, Shanghai, China | Cat # B802465 |
| Gingerenone A | Macklin, Shanghai, China | Cat # G922542 |
| Ferulic acid | Macklin, Shanghai, China | Cat # F809521 |
| Trans-cinnamic acid | Macklin, Shanghai, China | Cat # C804992 |
| Quercetin | Acmec Biochemical, Shanghai, China | Cat # M28790 |
| Cinnamaldehyde | Acmec Biochemical, Shanghai, China | Cat # C69860 |
| Gallic acid | Acmec Biochemical, Shanghai, China | Cat # G69650 |
| Glycyrrhizic acid | Acmec Biochemical, Shanghai, China | Cat # G75150 |
| Curcumin | Acmec Biochemical, Shanghai, China | Cat # C50900 |
| Sodium houttuyfonate | TargetMol, Shanghai, China | Cat # TN5284 |
| Allicin | TargetMol, Shanghai, China | Cat # T3123 |
